# A Methodology Based on Machine Learning and Soft Computing to Design More Sustainable Agriculture Systems

**DOI:** 10.3390/s23063038

**Published:** 2023-03-11

**Authors:** Jose M. Cadenas, M. Carmen Garrido, Raquel Martínez-España

**Affiliations:** Department of Information and Communication Engineering, University of Murcia, 30100 Murcia, Spain

**Keywords:** sustainable agriculture, time series forecast, Soft Computing, machine learning, IoT

## Abstract

Advances in new technologies are allowing any field of real life to benefit from using these ones. Among of them, we can highlight the IoT ecosystem making available large amounts of information, cloud computing allowing large computational capacities, and Machine Learning techniques together with the Soft Computing framework to incorporate intelligence. They constitute a powerful set of tools that allow us to define Decision Support Systems that improve decisions in a wide range of real-life problems. In this paper, we focus on the agricultural sector and the issue of sustainability. We propose a methodology that, starting from times series data provided by the IoT ecosystem, a preprocessing and modelling of the data based on machine learning techniques is carried out within the framework of Soft Computing. The obtained model will be able to carry out inferences in a given prediction horizon that allow the development of Decision Support Systems that can help the farmer. By way of illustration, the proposed methodology is applied to the specific problem of early frost prediction. With some specific scenarios validated by expert farmers in an agricultural cooperative, the benefits of the methodology are illustrated. The evaluation and validation show the effectiveness of the proposal.

## 1. Introduction

The role of technology in agriculture has particular relevance in the improvement of agriculture. A complete scheme of activities related to technology in agriculture is presented in [1], where authors analyse and identify sectors that can take advantage of the latest advances in technology. Using new technologies, farmers can monitor their farms remotely and more accurately, and therefore, they are considered an essential tool for sustainable agriculture. Sustainable agriculture is a concept that has become very important today. To be sustainable, agriculture must meet the food needs of present and future generations at prices that are reasonable for consumers and sufficient to maintain the economy of the agricultural sector without endangering the health of the environment or the quantity of natural resources. Sustainable agriculture is a system of agricultural production that is resource-conserving, environmentally sound, and economically viable. The aim is to produce healthy food with respectful practices for the soil, air, and water and to respect the rights and health of farmers.

Sustainable agriculture faces problems such as fertility loss, water scarcity, biodiversity depletion, pesticide pollution, climate change, and how it affects agriculture. In particular, climate change is causing major losses in agriculture, both in terms of product quality and farmers’ economies. Agricultural insurance is becoming increasingly restrictive and costly. Thus, farmers have to look for new solutions to mitigate crop damage without investing large amounts of economic and/or environmental resources.

Sustainable agriculture, and specifically precision agriculture, is considered to be an area of major interest within the objectives of sustainable development. Precision agriculture is a promising task for economic stabilisation in developing countries [2] and plays an important role in making agriculture more sustainable. It tries to measure crop qualities, soil, and climatic factors to apply the best treatment at the right place at the right time.

Precision agriculture is a discipline that began to be implemented in the early 1990s with the initial objective of increasing profitability and reducing environmental impact by using integrated systems within information technologies [3]. Every day more and more agricultural processes are beginning to have and use technology to improve their procedures, reducing economic and environmental costs and increasing profits, achieving a more sustainable agriculture [4]. In the process of adapting and contributing to sustainability, farmers need real and reliable sources of information and knowledge to make decisions more sustainable. Advances in technology and computational paradigms provide farmers with new methodologies to improve their crops [5].

Among these paradigms, IoT and cloud computing have become very relevant nowadays [6], making a large amount of information available to users in any real environment such as smart homes [7], the industrial market [8], and agriculture [9] among other areas. In the agricultural world, IoT and computing have revolutionised the sector, applying technologies to achieve service upgrades, cost savings, increased production, and better control and balance between economic and environmental sustainability [10]. In traditional agriculture, farmers have made decisions based on their experience, and although often right, at other times, both economic and environmental resources are wasted. Precision farming, coupled with computing and IoT paradigms, has improved these processes and reduced the economic and environmental impact. For example, in terms of spraying against pests [11], traditional farmers sprayed in a preventive way with highly polluting products without knowing the level of pests they had in their crops. Now, there are crop pest prediction techniques that help to spray at the right time and with the right products [12]. Thus, now with precision farming, the farmer saves on the cost of crop protection products, and the environment gains in terms of less pollution. Another example of improving sustainability with new technologies is irrigation control. By predicting temperatures, as well as predicting soil moisture levels, the amount of water used on crops can be reduced. Again, new technologies help to achieve economic sustainability in the reduction of water costs and environmental sustainability in the use of water [13].

Thus, new technologies help the agricultural sector to be more sustainable. However, many of the proposed techniques are not deployed in real environments, and one of the problems is the lack of a methodology that includes the steps and the way to implement the new technologies in agricultural problems. Hence, the novel proposal of this paper develops a generic methodology, which can be applied to agricultural problems, with the aim of having a final Decision Support System to make decisions with implications for economic and environmental improvement. To build a Decision Support System, it is necessary to have data. The IoT poses through all kinds of sensors to collect and send data related to farmland. This information collected by sensors, and depending on the scope of the application, can contain numerical values following the trend of a time series. This indicates that there are dependencies on one value, the next, and the previous one. The collected information generates significant amounts of data that can be analysed remotely and in real-time using appropriate Machine Learning techniques. With the use of these techniques, patterns can be found from the collected data, and predictions can be made, providing intelligence to the Decision Support Systems. Studies have shown that Decision Support Systems applied to agriculture contribute significantly to long-term sustainable development. However, the full benefits of Decision Support Systems are not yet realised, as they need to be adapted to the needs of farmers [4].

This paper proposes the design of a novel methodology aimed at facilitating the creation of a Decision Support System to address agricultural problems. The final system will be embedded in a mobile device where usability is simple, transparent, and flexible for the farmer. In this way, the farmer can benefit from the use of applications with specialised information about crops and techniques to improve decision-making in crop management, changes in their production systems, etc. The choice of the mobile phone as the final device is based on the fact that there are studies that indicate that farmers are using their smartphones more as work tools than for entertainment and that they prefer to access the Internet using their mobile phone rather than other devices [14].

As the main characteristics of the methodology, we can highlight that it is general to address agricultural problems to create a Decision Support System using computing and IoT paradigms, is independent of particular techniques and technologies, is designed to address problems that improve the environmental and economic sustainability of farmers, and can deal with one of the disadvantages of the IoT, which are precision errors in sensors and possible delivery failures. The methodology is embedded in the Soft Computing framework that allows dealing with these problems using a representation of data that captures the true nature of them.

The methodology takes as a starting point the information collected in the IoT ecosystem in the form of time series data, and its ultimate goal is the creation of a Decision Support System embedded in a mobile phone. In a general way, the methodology is defined based on the following stages: (1) obtaining data from IoT and cloud services, (2) preprocessing the data in the Soft Computing framework, (3) extracting knowledge using appropriate Machine Learning techniques, and (4) defining a set of rules that constitute the main axis of the Decision Support System.

The study developed in this paper is organised as follows: Section 2 presents a non-exhaustive review of proposals for solving real problems related to sustainable agriculture from time series data that can obtain advantages of the design of a general methodology. Section 3 details the proposed methodology by presenting each of its stages. Section 4 presents a study case where the proposed methodology is applied step by step to the crop frost problem, illustrating both the environmental and economic benefits obtained in terms of sustainability. Finally, Section 5 shows the conclusions of the study and future work lines.

## 2. Background

As mentioned above, the role of technology in agriculture has particular relevance in improving agriculture. The use of paradigms such as machine learning and Soft Computing can help the development of sustainable agriculture by providing the information obtained through IoT with Artificial Intelligence [15]. The joint use of these technologies helps the design of Decision Support Systems that lead to the development of sustainable agriculture. Within this field, these technologies have been used, among others, in the prediction of adverse weather conditions, optimisation of water resource management, optimisation of crop productivity, etc.

In this section, we review, without being exhaustive, some studies that try to introduce benefits in agriculture to make it more sustainable from the information provided by time series datasets collected from the IoT ecosystem and the use of Artificial Intelligence paradigms. The aim is to show the wide range of possible benefits rather than to go deeper into any of them.

As a consequence of climate change, the construction of Decision Support Systems that help farmers to be aware of the arrival of possible adverse weather phenomena such as frost, floods, etc., is getting more and more important. The problem arises because weather phenomena are becoming more frequent, according to experts [16]. Thus, to address these problems, we can find various works in literature. In this way, in [17], the authors use time series datasets to predict short/long term low temperatures by capturing the dependencies of environmental factors through causal and associative models. In [18], frost prediction is carried out in advance using deep learning techniques using a long short/term memory model. In [19], models of the rainfall are obtained using deep learning architectures and time series datasets to minimise the risks caused by rainfall and reduce the economic risks and, therefore, maximising profits.

As it is well known, water is a vitally important resource in agriculture and has been considered one of the critical issues in the future that may threaten food security. For this reason, studies have been carried out to measure the quality of the water and optimise the quantity of water used for crops. For example, in [20], the quality of drained water that is reused for agriculture is studied using neural network models that will help to manage the resource and make decisions about irrigation water resources. An important factor in managing the water resource in crop production is the accurate prediction of evapotranspiration. The control of this factor can optimize the amount of water needed. To address this control, in [21], the authors present the development of a computational method for estimating the monthly mean evapotranspiration of arid and semi-arid areas is carried out.

Another of the most important issue in agriculture is improving crop management. In this sense, yield prediction will enable the farmer to improve crop management to match crop supply to demand and increase productivity. In the same way, the productivity and quality of crops can be improved by detecting the appearance of diseases at an early stage and carrying out a more appropriate treatment. Thus, in [22], the authors carry out the wheat yield production from satellite images to receive measurements of crop and soil growth. In [23], a classification of the leaves of several plants is carried out to distinguish between healthy and diseased leaves based on a neural network and images of the leaves. Other work is presented in [24] where the detection of diseases in wheat is carried out to optimise the use of fertilisers and fungicides according to the needs of the plant.

Regarding the right estimation of crop soil conditions improves soil management by the farmer. Nevertheless, the obtaining of soil measurements is generally expensive and time-consuming, and the process can be improved by using Machine Learning techniques. Some works in this line are [25,26]. In the first one, soil drying is evaluated from evapotranspiration and rainfall data to favour decision-making in crop planning. In the second one, the authors develop a Machine Learning technique to obtain soil temperature at different depths with the aim of optimising soil management.

As a summary, many agricultural domains can obtain benefits from using new technologies, and the methodology proposed in this work defines a series of steps to follow to achieve this objective.

## 3. A Methodology Based on IOT Ecosystem and Machine Learning in the Soft Computing Framework

New developments in IoT and cloud computing technologies enable applications to have a wealth of information from any domain in real-time. Depending on the application domain, the information comes in consecutive time intervals resulting in time series datasets. The information can be processed, and knowledge can be obtained from it using Machine Learning techniques; however, before using such techniques, it is necessary to apply preprocessing to the data [27]. There are many data preprocessing tasks, but the most common are: using the Fourier transform to shift the series to the frequency domain, using statistical values such as the mean to make an aggregate symbolic approximation, performing a transformation of the values to depict the time evolution, etc.

This section details the proposed methodology for preprocessing time series datasets to be used in Machine Learning techniques. As a result of applying the methodology, a new dataset that can be used efficiently by Machine Learning techniques is created while the quality of the original information is maintained. The model obtained after applying the Machine Learning techniques will be the base of the designed Decision Support System.

The main stages that make up the methodology are as follows:

1. Data collection. The starting point of the methodology is the collection of data representative of the agricultural domain to be solved. This data collection will be carried out through the IoT ecosystem and cloud services. Therefore, the methodology is integrated into the FIWARE architecture.
2. Preprocessing of time series data. The processing of time series data will allow synthesising the number of attributes using the imprecise representation of an attribute or set of attributes without losing information. This will be carried out in the Soft Computing framework. Furthermore, the preprocessing uses lagging attributes to maintain the connection of the time series in the data.
3. Application of Machine Learning techniques. In this stage, the Machine Learning techniques used must be capable of dealing directly with imprecise information with two objectives. The first objective is to select the most important instances to build simple, useful, and portable models that can be used in embedded devices without an internet connection. The second objective is to obtain a useful model to solve the problem.
4. Obtaining the Decision Support System by defining a set of rules that allow the farmer to make a decision based on the model obtained in the previous stage and the data provided in real-time by the Context Orion Broker of the FIWARE architecture.

**Figure 1:**
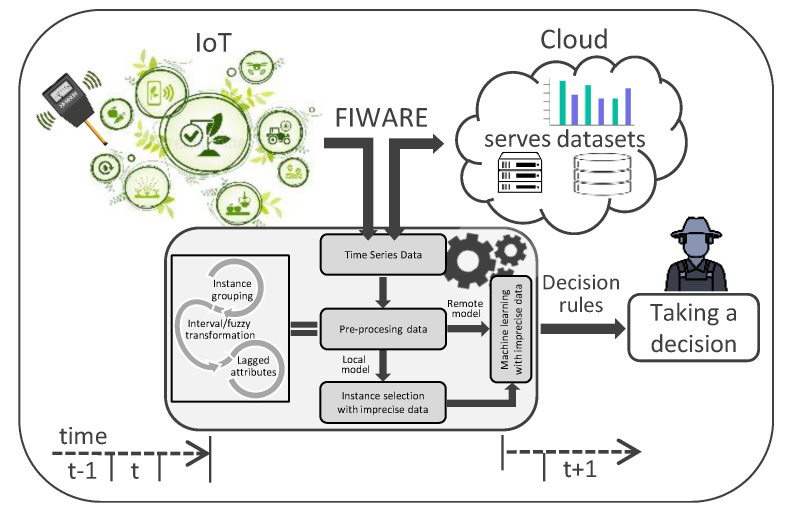
Proposed methodology embedded within an IoT ecosystem.

**Figure 2:**
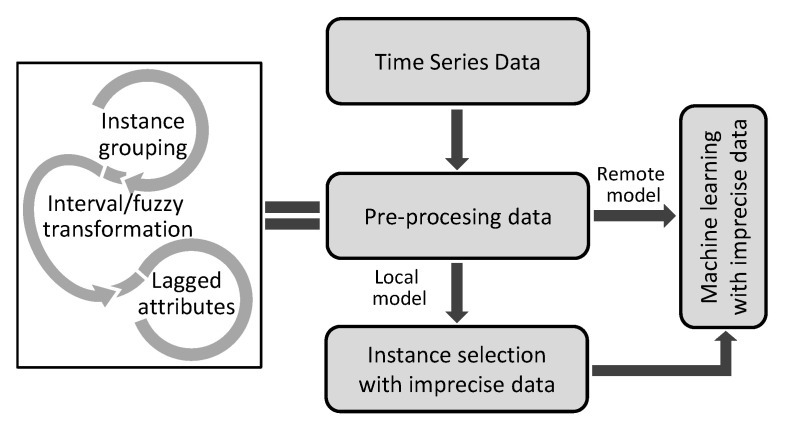
Diagram of preprocessing and model building.

IoT ecosystem integrating the proposed methodology has the structure shown in Figure 1. The stages describing the preprocessing and construction of the model are shown in Figure 2.

Each stage of this methodology is detailed in the following subsections.

### 3.1. Data Collection

As mentioned above, the source of information is the IoT ecosystem and cloud services that will form part of the methodology in two clearly differentiated areas:

- Collection of data from the IoT ecosystem and cloud services stored persistently. The aim is to obtain information stored in datasets from different context providers in the field of agriculture, such as different types of weather station sensors. It is, therefore, about historical data that will be used, after the appropriate preprocessing, to obtain Machine Learning models that allow the construction of Decision Support Systems. This historical data will comprise successive measurements made by the different context providers in certain time intervals. Therefore, there is a temporal dependence between the different instances that comprise the dataset. The use of this data will be carried out offline.
- Obtaining data from context providers in real-time. Once the Decision Support System has been built, it is necessary to obtain real-time data from the different context providers to make an appropriate decision according to the scope of the application. For example, if the application is designed to monitor the quality of a crop soil, the data obtained in real-time from the soil properties could generate an alert to farmers indicating that a certain threshold of shortage of fertility properties of their crops, such as pH, salinity, and available plant nutrients has been reached. The use of this data will be carried out online.

The designed application will be integrated into the FIWARE architecture. The FIWARE architecture consists of several components stored on a server. This server is responsible for storing the information received from the sensors and different web services provided by opendata, among others. In addition, the server is also responsible for making the data available to the visualisation services in the user interfaces as well as providing data to the intelligent data analysis module. The FIWARE architecture interacts with the mobile application through a BackEnd service located on the server that connects the application with the Orion Context Broker. The Orion Context Broker is a FIWARE component that is responsible for providing the data corresponding to the mobile application, this being a transparent process to the user. This component also connects with the framework services that collect the data, being weather stations or farmers’ own sensors or open data provided by meteorological services, agricultural companies, or official information services, among others. The information collected from the different sources is stored using a database, which can be structured like MySQL or unstructured like MongoDB, or a combination of both. The information is received using JSON data type protocols.

### 3.2. Preparing the Dataset

#### 3.2.1. Instance Grouping

When working with time series datasets, it is common to find datasets large in data size and with high dimensionality as these datasets store the complete set of data provided by the different measurement processes (in general, data continuously provided by sensors). There are different proposals to deal with this problem [28].

The methodology, we propose, faces the problem of large data volume by performing a clustering of instances that sometimes does not only solve the problem of data volume but can be directly driven by the application. This clustering combines data into certain intervals like each hour, each day, a week, or a month. The size of the clustering will depend on the application.

**Figure 3:**
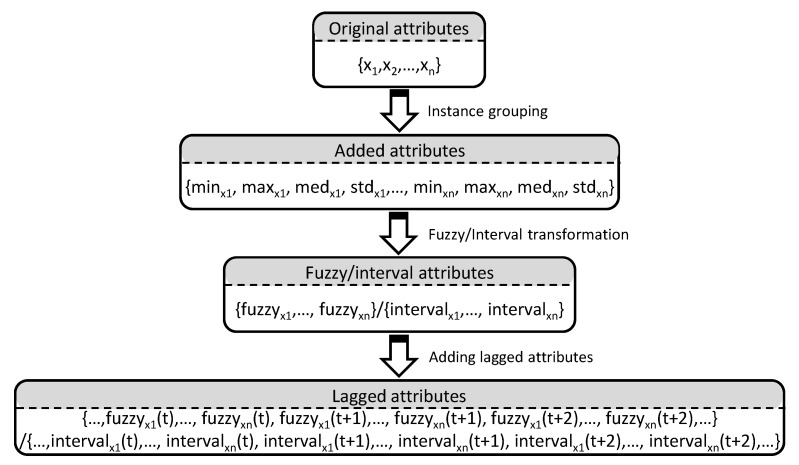
Methodology for preprocesing data.

Therefore, in the proposed methodology, as a first preprocessing step, a clustering of instances will be carried out. For this purpose, a certain number of consecutive instances are grouped together, and information representative of this grouping is added to the dataset in the form of new attributes such as minimum, maximum, mean, and standard deviation values, among others. Therefore, while this clustering leads to a reduction in the volume of data (number of instances), it also leads to an increase in the number of attributes describing each time series, and this can lead to an increase in dimensionality. This problem of increased dimensionality will be solved in the following stages of the methodology. This increase in dimensionality becomes more considerable in problems that require long-term forecasting, as we will see later. The effect of clustering instances is illustrated in the first step of Figure 3.

#### 3.2.2. Interval/Fuzzy Transformation

The increase in the number of attributes in the dataset can be addressed by grouping the attributes with crisp values associated with the same time series with attributes with interval or fuzzy values. In these interval/fuzzy attributes, the information is captured, and no loss of information occurs. Thus, there is no increase in the dimensionality of the data. Figure 3 shows the original attribute clustering process.

Given that this methodological proposal involves incorporating attributes expressed through fuzzy values, the following phases of the methodology will require the use of Machine Learning techniques capable of dealing with this type of values, i.e., techniques defined in the Soft Computing framework. The fact of using this type of technique also allows us to carry out the treatment of missing data without making a great effort in the methodology.

Missing values are one of the problems frequently encountered in the observation or data recording process. It is important to address the need for completeness of the observation data to be able to use it for advanced analyses. Conventional methods, such as mean and mode imputation, elimination, and other strategies, are not good enough to deal with missing values, as they may cause biases in the data [29]. In [30], it is proposed to treat the missing values by expressing them as fuzzy values reflecting their true nature and using the appropriate techniques for this type of value. Therefore, the data will be completed and ready to use for another step of analysis or data mining.

In addition, the use of techniques capable of dealing with fuzzy values also allow the possibility of working with time series datasets that incorporate nominal attributes. In this case, each nominal attribute will be replaced by a discrete fuzzy attribute that summarises the possibility that each value of the domain of that nominal attribute. Thus, given the nominal attribute *A* with domain $\Omega_{A}=\left\{v_{1},v_{2},\ldots ,v_{n}\right\}$, this attribute will be replaced by a fuzzy attribute expressed by the membership function $\mu_{A}=\left\{v_{1}/\mu_{1},v_{2}/\mu_{2},\ldots ,v_{n}/\mu_{n}\right\}$.

#### 3.2.3. Adding Lagged Attributes

Time series datasets are used to predict the value of some attribute at a future time instant t+1 from past values. In the proposed methodology, the time series dataset is transformed by including lagged attributes.

Therefore, after the above transformations, the temporal correlation between consecutive instances of the dataset is captured by adding delayed attributes. These added attributes allow us to capture a prediction horizon that will depend on the problem we are dealing with. Thus, we will group in the same instance as many delayed attributes as the size of the prediction horizon. For example, in a problem with hourly information and a prediction horizon of “*x*” hours, we will have to add “*x*” delayed attributes that capture the temporal correlation between hours…,h−1,h,h+1,…. Depending on the specific application in which the methodology is being applied, the prediction horizon can be longer or shorter, and we can talk about short-term time series or long-term time series.

In either case, the inclusion of lagged attributes increases the dimension of the problem, so the reduction of attributes carried out by the fuzzy transformation allows for the reduction of this dimension while capturing the true nature of the information. The effect of this step of the methodology is shown in the last step of Figure 3.

### 3.3. Using Machine Learning Techniques

After carrying out the previous preprocessing process, the objective now is to obtain a model of the data through the use of Machine Learning techniques that are capable of working with imprecise data. This model will form part of the final Decision Support System that will bring to the farmer the advantages and benefits provided by the Artificial Intelligence research field and, more specifically, by Machine Learning and Soft Computing. In this way, any farmer with a smartphone with or without an internet connection can benefit from the advances in these disciplines.

We are, therefore, considering two ways of working with the methodology:

- Local mode: No internet connection to address the problem of lack of wireless signal coverage that can easily occur in rural areas. In this case, the Decision Support System will be hosted on the smartphone. In this working mode, it is important that the data model has a space and time complexity optimised execution. For this reason, in this working mode, it is proposed in the methodology to add an instance selection process that again reduces the size of the set of instances with which the Machine Learning technique will work.
- Remote mode: With internet connection. In this case, the Decision Support System will be hosted on a cloud server and although time and space complexity are always important, it is not crucial in this way of working.

These modes of work are reflected in the working scheme in Figure 2.

#### 3.3.1. Instance Selection Process

In the case of working in local mode and to optimise the size of the data model, the next step of the proposed methodology is to apply an instance selection process. The instance selection technique has to be a preprocessing technique able to handle heterogeneous attributes (nominal and numerical) with imprecise and crisp values. Among these techniques, we can use the CNN technique [31] with the kN$N_{imp}$ technique [30] to classify each instance and add it to the condensed dataset if it is not correctly classified with the current condensed set. kN$N_{imp}$ technique allows the treatment of imprecise data for both classification and regression. In general, we can use any instance selection technique based on kNN nearest neighbours that takes as a basis the kN$N_{imp}$ technique that allows the treatment of imprecise data.

#### 3.3.2. Machine Learning Model

The Machine Learning model is the one that will be in the application so that the user can obtain answers to the problem addressed. The model may be locally on the device or stored in the cloud, depending on the type of mode (local, remote) that the user will use. In addition, the techniques that create such a model in this methodology should be classification or regression techniques that can handle heterogeneous attributes and imprecise and crisp values. Among the techniques available to perform the classification/regression task with imperfect data we can find kN$N_{imp}$ [30], Fuzzy Random Forest, and FDT [32].

### 3.4. Decision Support System

Once the Machine Learning model has been obtained, it is necessary to integrate it into the Decision Support System that helps the user to make decisions in the application environment. A general and intuitive way to build this Decision Support System is by defining a set of rules that will be activated according to the output of the obtained model and the real-time data provided by the IOT ecosystem through FIWARE.

In general, the rules will be of the form: *if condition then action*. This set of rules will depend on the domain of the specific application being developed and must gather the knowledge of the domain expert, the farmer, to define the behaviour of the rules, e.g., the definition of which threshold values should define the activation of a rule (condition) and the achievement of the action derived from this activation (action).

In the case of using a classification technique, it will be the value of the inferred class that determines the action to be taken with rules of the form:
if class1 then Action1if class2 then Action2…


In the case of using a regression technique, the use of threshold values in the scope of application will determine rules of the type:
if value ≤ threshold then Action1if value > threshold then Action2

## 4. Study Case: Early Crop Frost Forecasting

In this section, we are going to focus on a specific problem where farmers could benefit economically as well as improve the sustainability of their crops. Specifically, we will follow the steps of the methodology to obtain a Decision Support System that, by means of an early temperature prediction, will help farmers to make good decisions regarding frost management. The final system will be validated by expert farmers advised by an agricultural cooperative.

In the southeast of Spain, there are areas where temperatures from January to March vary greatly. These temperatures reach warm values during the day but reach values below zero during the night. These sudden changes in temperature produce harmful effects on crops. In this area of Spain, where a large part of the crops are dedicated to fruit trees, the daytime heat produces the flowering of the crops while the cold at night destroys this flowering. However, farmers have various techniques to mitigate the effects of frost. These techniques include thermal mulching to cover crops, sprinkler irrigation using the heat released during icing, creating artificial fog, and heating cold air close to the ground by burning several kinds of fuels [33].

This fight against frost needs an early and realistic prediction of the temperature forecast for the immediate future to have enough time to deploy the techniques (to try not to lose the crop) and because the use of these techniques represents a significant additional cost that adds to the expenses faced by a farmer. This is where farmers can benefit from the methodology proposed in this paper by taking advantage of the early forecast of the temperature that will be reached on their land.

Therefore, the objective of this study case is to predict the temperature one hour in advance based on information obtained from weather stations that are close to the crops and are part of the IoT ecosystem.

### 4.1. Data Collection/Obtaining

As we have just mentioned, the information we are interested in this study case is obtained from the IoT ecosystem by taking the weather stations closest (denoted by CW$S_{i}$) to 5 crops from different geographical areas of the Region of Murcia as context providers. These weather stations are provided by The Murcian Institute of Agricultural and Food Research and Development (IMIDA) [34].

The IMIDA’s weather stations have the following ephemeris and sensors: rain gauge, radiometer, data-logger, weather vane, and thermohygrometer. The obtained measures are the following: “Radiation”, “Accumulated radiation”, “Rainfall”, “Humidity”, “Temperature”, “Wind direction”, “Wind speed”, “Dew point”, and “Vapor pressure deficit”. The IMIDA records these values every 5 minutes and therefore, this stored information gives rise to the time series datasets that are used as starting point in this study case. A time series dataset is obtained for each weather station.

### 4.2. Instance Grouping

As the objective is to carry out the prediction of the “Temperature” one hour later, we are going to group the data obtained from each station 12 by 12 so that each instance of the transformed dataset gathers information from one hour. To not lose information in this grouping, those measurements with a significant variation during the course of the hour are replaced by three attributes containing the minimum, mean, and maximum values obtained during that hour. This is what happens with the measures “Radiation”, “Humidity”, “Temperature”, and “Wind speed”.

The attributes obtained after grouping are shown in Table 1.

In addition, in the datasets obtained after grouping, the instances with the attribute “Minimum temperature” >7 are deleted as they are not representative of the problem.

### 4.3. Interval/Fuzzy Transformation

After grouping instances, the number of attributes of the time series dataset is increased (from 9 attributes to 17) not to lose information. This increase in dimensionality can be decreased by the fuzzy transformation of the proposed methodology. Those attributes that correspond to the minimum, average, and maximum values of the same measure are represented by a fuzzy attribute that expresses the true nature of the information provided by these attributes. The fuzzy attributes collecting the minimum, mean, and maximum attributes for a measure are those shown in Table 2 with the subscript *f*.

The fuzzy attributes are expressed by a trapezoidal function defined by the values $\left[x_{1},x_{2},x_{3},x_{4}\right]$. More concretely, the constructed fuzzy attributes are defined by the following values: $x_{1}=minimum$, $x_{2}=mean-5\%\;mean$, $x_{3}=mean+5\%\;mean$, and $x_{4}=maximum$, where minimum, mean, and maximum are the three values available for the same measurement. As commented above, incorporating fuzzy values in the datasets allows us to incorporate also the treatment of missing values expressed as fuzzy values as $\left[minimum_{dom},minimum_{dom},maximum_{dom},maximum_{dom}\right]$ where $minimum_{dom}$ and $maximum_{dom}$ are the extremes of the domain of the attribute. This representation of a missing value expresses that it can be any value in the interval.

### 4.4. Adding Lagged Attributes

**Figure 4:**
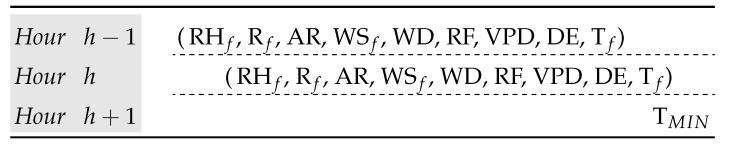
Attributes that define a final instance in the dataset. This instance captures the temporal relationship between three consecutive hours.

In this preprocessing step, the time dependence of the dataset is taken into account adding lagged attributes. The number of lagged attributes to be added will depend on the size of the prediction horizon to be carried out and the particular problem being solved. In this study case, since the objective is to forecast the temperature one hour in advance, an instance is formed by the values obtained for the set of attributes of Table 2 in the hour h−1, those obtained in the hour *h*, and the value of the minimum temperature in the hour h+1. Therefore, from the historical dataset collected from a weather station, we obtain the final dataset by joining the values of two consecutive instances and the attribute “Min. Temperature” ($T_{MIN}$) of the next instance. The structure of the final instance in the dataset is shown in Figure 4.

Obviously, we could add a higher number of lagged attributes but it is important to maintain a balance between the accuracy of the model obtained and the size of the dataset. The choice of the number of lagged attributes is an adjustment to be made at the preprocessing step.

As a final result of this preprocessing step, we obtain a dataset for each of the 5 weather stations considered in this study case. The characteristics of each of them are shown in Table 3 where *I* is the number of instances of the dataset, Na the number of attributes, Nm and Nf are the percentage of missing and fuzzy values respectively, and Nmf is the percentage of instances with missing and/or fuzzy values. We use trapezoidal fuzzy numbers to represent the fuzzy attributes. This representation is reflected in a vector of four values of the form [$a_{1}$, $a_{2}$, $a_{3}$, $a_{4}$] where $a_{i}$ are values of the measured attribute domain, $a_{1}\leq a_{2}\leq a_{3}\leq a_{4}$, and $a_{1}$, $a_{4}$ have a membership degree of 0, and $a_{2}$, $a_{3}$ have a membership degree of 1.

### 4.5. Instance Selection Process

This step of the methodology allows us to reduce the set of instances while maintaining the accuracy of the predictions made by the model. This process is particularly important when we want to use the model in local mode and we are going to work with a Machine Learning technique of the “Lazy learning” category [35] where the model is made up of the data itself.

To perform instance selection on the datasets of Table 3, it is necessary to use a technique that allows dealing with fuzzy and missing values. In this study case, we will use the CNN technique, as we commented in Section 3.3.1, to deal with imprecise values. Since the technique needs a class attribute, we add (only for the instance selection process) to each instance a class value from the set {*F*,NF} where the instance is labelled with the value NF if $T_{min}>0$ or with the value *F* in otherwise. This technique ranks the instances in increasing order of their imprecision so that the more imprecise instances are, a lower probability of belonging to the final reduced set will have.

The best model in terms of size and accuracy has been included in the Decision Support System. The model has been set with the parameter k=1 and obtains to each dataset size of 1962, 1488, 1819, 999, and 664 instances (with a percentage of reduction of 87.08%, 87.45%, 80.00%, 88.20%, 92.00%), respectively.

### 4.6. Machine Learning Model

To design the Decision Support System, a Machine Learning technique is needed to predict the temperature one hour in advance. This technique must be able to work with imprecise data. In this study we use the kN$N_{imp}$ technique [30]. As a result of the previous analysis, the kN$N_{imp}$ will work with the instances included in the condensed datasets with k=1 for each station.

### 4.7. Decision Support System

In this study case, and after consulting the farmer about the number of rules, the threshold values, and the actions to be displayed, the designed set of rules is as follows:

$$\text{If}\;2\;<\;T_{\min}\;\leq 5\;,\;\mathrm{then}\;\mathrm{send}\;“\mathrm{Alert}—\mathrm{Very}\;\mathrm{low}\;\mathrm{temperature}\;\mathrm{forecast}”\;\mathrm{to}\mathrm{farmer}\;\mathrm{If}\;T_{\min}\;\leq 2\;\mathrm{then}\;\mathrm{send}\;“\mathrm{Alarm}—\mathrm{Frost}\;\mathrm{forecast}\;\mathrm{soon}”\;\mathrm{to}\mathrm{farmer}$$

where *T_min_* is the forecast of the temperature value for the next hour. Therefore, the farmer will receive an alert/alarm that will allow him/her to take the necessary measures to protect his/her crops one hour in advance.

### 4.8. Evaluating the Decision Support System by Expert Farmers

When the system is in service, it collects the values of the attributes (R$H_{f}$, $R_{f}$, AR, W$S_{f}$, WD, RF, VPD, DE, $T_{f}$) in the current hour *h* and the previous one h−1 and obtains a prediction of T for hour h+1. For example, at hour 21:00, the collected information is ([71.62, 73.55, 74.85, 75.91], 0.0, 0.0, [0, 0.37, 0.73, 1.27], 347.5, 0.0, 0.24, 1.39, [4.98, 5.45, 5.78, 6.27]) and at hour 22:00 is ([74.58, 77.32, 79.15, 80.80], 0.0, 0.0, [0, 0.23, 0.99, 2.16], 338.3, 0.0, 0.18, 0.72, [3.28, 3.92, 4.41, 5.15]).

Table 4 shows the predictions made by the Decision Support System in three different time scenarios. The table shows the time at which the prediction is made and the time for which the temperature value is predicted.

The three scenarios in Table 4 have been validated by two farmers from “Sociedad Cooperativa Alimer Alimentos del Mediterráneo” (https://alimer.es/ accessed on 30 January 2023) in the Region of Murcia. The farmers have paraffin burners on their plot as an anti-frost system. On the one hand, if the farmers would not have the prediction system, and according to the current temperature values in period 1 (Table 4), the farmers indicate that at 23:00, they would have been alert about the activation of the anti-frost system (devices, staff, etc.). At 24:00, they would have activated the system and kept it until 10:00, when they would have deactivated it. On the other hand, with the information provided by the system on the temperature prediction one hour in advance (Table 4), the farmers indicate that at 22:00 and also knowing the prediction for 23:00, they would be on alert, at 23:00 with the prediction for 24:00, they would remain on alert, and so on until 01:00. At that time they know that at 02:00 the temperature will be below $1^{{}^{\circ}}$ positive and therefore, at 01:00 they would start preparing their anti-frost system to activate it at 2:00. At 09:00 they know that the forecast for 10:00 will be over $2^{{}^{\circ}}$ positive and therefore, from 09:00 they would start to deactivate their anti-frost system. Thus, with the Decision Support System created using the proposed methodology, the farmers have saved two hours of both economic and environmental resources.

In period 2, and with the information shown in Table 4, farmers act similarly to period 1, but in period 3 (Table 4) we must highlighted an interesting situation. The farmers tell us that if they would not have the forecast, and with the current values, at 04:00, they would have activated the anti-frost system. However, with the forecasting system, at 04:00 they know the forecast for 05:00, and, therefore, at 05:00, they would be on alert and would make their decision to activate the anti-frost system based on the forecast for 06:00. As at 5:00 the forecast for 06:00 remains unchanged, and because of the night is near to end, they would decide not to activate the system, waiting for the next hour. Thus, in the end, and with the information available, the farmers would not activate the anti-frost system.

As we can see, with the information provided by the prediction system in these three situations, farmers make more appropriate decisions and, therefore, can reduce the activation time of the anti-frost system, or not activate it at all. This leads to savings both in terms of the devices used and the staff required to activate them and reduces the environmental impact involved.

## 5. Conclusions and Future Work

This paper has presented a methodology to develop Decision Support Systems that help to make decisions in the field of agriculture. The methodology is based on time series datasets, and after applying its different stages, an inference engine is obtained that provides the final system with intelligence. In an encapsulated form, it allows farmers to benefit from new technologies and to be less reluctant to use them in their work. A study case for the crop frost problem has been shown in the paper. In that study, the proposed methodology is used, offering a warning solution to farmers so that they can prevent crop frost. The final system has been validated by experts from an agricultural cooperative, showing its effectiveness in terms of economic and environmental benefits. Therefore, the objective pursued with the methodology has been achieved.

The methodology can be extended and customised to use in other areas of today’s world. Furthermore, it would benefit from the development and research of new Machine Learning techniques in the Soft Computing framework or the improvement of existing ones.

## Figures and Tables

**Table 1:** Instance attributes after grouping the data of each hour.

Instance Attributes	
Minimum relative humidity (%)	Mean relative humidity (%)
Maximum relative humidity (%)	Minimum radiation (W/$m^{2}$)
Mean radiation (W/$m^{2}$)	Maximum radiation (W/$m^{2}$)
Accumulated radiation (W/$m^{2}$)	Wind direction (°C)
Minimum wind speed (m/s)	Mean wind speed (m/s)
Maximum wind speed (m/s)	Rainfall (mm)
Vapor pressure deficit (kPa)	Dew point (°C)
Minimum temperature (°C)	Mean temperature (°C)
Maximum temperature (°C)	

**Table 2:** Fuzzy transformation of attributes.

Transformed Attributes	Description
R$H_{f}$	Fuzzy relative humidity
$R_{f}$	Fuzzy radiation
AR	Accumulated radiation
W$S_{f}$	Fuzzy wind speed
WD	Wind direction
RF	Rainfall
VPD	Vapor pressure deficit
DE	Dew point
$T_{f}$	Fuzzy temperature

**Table 3:** Description of the five datasets in the studio case.

Name	*I*	*Na*	*%Nm*	*%Nf*	*%Nmf*
CW$S_{1}$	15,185	19	0.02	44.4	100
CW$S_{2}$	11,855	19	0.01	44.4	100
CW$S_{3}$	9094	19	0.02	44.4	100
CW$S_{4}$	8470	19	0.01	44.4	100
CW$S_{5}$	8296	19	0.01	44.4	100

**Table 4:** Temperature prediction for the next hour (h+1) from the current information (*h*) and the previous one (h−1). The information for the time *h* and h−1 corresponds to the vector (R$H_{f}$, $R_{f}$, AR, W$S_{f}$, WD, RF, VPD, DE, $T_{f}$). The time at which the prediction is made, the predicted temperature one hour in advance, and the real values that occurred, are indicated.

	Prediction Made…				Real	
	**At**	**For**	**Expected Temperature**	**System Message**	**Hour**	**Real Temperature**
Period 1	21:00	22:00	4.200	“Alert—Very low temperature forecast”	22:00	3.281
	22:00	23:00	2.602	“Alert—Very low temperature forecast”	23:00	2.881
	23:00	24:00	2.359	“Alert—Very low temperature forecast”	00:00	1.525
	24:00	01:00	1.135	“Alarm—Frost forecast soon”	01:00	1.253
	01:00	02:00	0.726	“Alarm—Frost forecast soon”	02:00	0.776
	02:00	03:00	0.084	“Alarm—Frost forecast soon”	03:00	−0.075
	03:00	04:00	−0.426	“Alarm—Frost forecast soon”	04:00	−0.483
	04:00	05:00	−0.902	“Alarm—Frost forecast soon”	05:00	−0.886
	05:00	06:00	−1.327	“Alarm—Frost forecast soon”	06:00	−1.461
	06:00	07:00	−1.691	“Alarm—Frost forecast soon”	07:00	−1.594
	07:00	08:00	−1.957	“Alarm—Frost forecast soon”	08:00	−1.662
	08:00	09:00	−1.751	“Alarm—Frost forecast soon”	09:00	−1.457
	09:00	10:00	2.610	“Alert—Very low temperature forecast”	10:00	2.716
Period 2	22:00	23:00	5.730	“Non-Alert”	23:00	5.322
	23:00	24:00	4.510	“Alert—Very low temperature forecast”	24:00	3.965
	24:00	01:00	3.418	“Alert—Very low temperature forecast”	01:00	3.396
	01:00	02:00	2.657	“Alert—Very low temperature forecast”	02:00	2.818
	02:00	03:00	1.821	“Alarm—Frost forecast soon”	03:00	1.627
	03:00	04:00	0.875	“Alarm—Frost forecast soon”	04:00	1.525
	04:00	05:00	0.534	“Alarm—Frost forecast soon”	05:00	0.572
	05:00	06:00	−0.072	“Alarm—Frost forecast soon”	06:00	0.061
	06:00	07:00	−0.268	“Alarm—Frost forecast soon”	07:00	−0.206
	07:00	08:00	−0.269	“Alarm—Frost forecast soon”	08:00	0.444
	08:00	09:00	3.656	“Alert—Very low temperature forecast”	09:00	3.839
Period 3	01:00	02:00	3.926	“Alert—Very low temperature forecast”	02:00	4.033
	02:00	03:00	2.868	“Alert—Very low temperature forecast”	03:00	2.478
	03:00	04:00	2.170	“Alarm—Frost forecast soon”	04:00	1.865
	04:00	05:00	1.335	“Alarm—Frost forecast soon”	05:00	1.184
	05:00	06:00	0.743	“Alarm—Frost forecast soon”	06:00	1.116
	06:00	07:00	0.654	“Alarm—Frost forecast soon”	07:00	0.742
	07:00	08:00	1.069	“Alarm—Frost forecast soon”	08:00	1.457
	08:00	09:00	4.856	“Alert—Very low temperature forecast”	09:00	5.167
